# Cerebrospinal fluid matrix metalloproteinase 9 levels, blood-brain barrier permeability, and treatment outcome in tuberculous meningitis

**DOI:** 10.1371/journal.pone.0181262

**Published:** 2017-07-12

**Authors:** Sharada Mailankody, Gurukiran V. Dangeti, Rajendiran Soundravally, Noyal M. Joseph, Jharna Mandal, Tarun K. Dutta, Tamilarasu Kadhiravan

**Affiliations:** 1 Department of Medicine, Jawaharlal Institute of Postgraduate Medical Education and Research, Puducherry, India; 2 Department of Biochemistry, Jawaharlal Institute of Postgraduate Medical Education and Research, Puducherry, India; 3 Department of Microbiology, Jawaharlal Institute of Postgraduate Medical Education and Research, Puducherry, India; Albert-Ludwigs-Universitat Freiburg, GERMANY

## Abstract

**Objectives:**

Tuberculous meningitis is characterized by elevated levels of matrix metalloproteinase 9 (MMP9) in the cerebrospinal fluid (CSF). However, it is unclear whether elevated MMP9 levels are associated with poor treatment outcome. We tested the hypothesis that pretreatment MMP9 levels in the CSF would be higher in tuberculous meningitis patients experiencing a poor treatment outcome.

**Methods:**

We prospectively assessed the treatment outcome in a consecutive sample of human immunodeficiency virus-negative patients with tuberculous meningitis. We defined good outcome as survival without severe neurological disability (modified Rankin scale scores 0–2). We estimated levels of MMP9 and its tissue inhibitor (TIMP1) on pretreatment CSF samples. We used albumin index to assess blood-brain barrier permeability.

**Results:**

We studied 40 patients (23 males [58%]) with tuberculous meningitis. Sixteen patients (40%) had stage 3 disease. On follow-up, 18 (45%) patients had a poor treatment outcome—15 patients died and 3 had severe neurological disability. Pretreatment MMP9 levels were not associated with treatment outcome (median [interquartile range], 254 [115–389] vs. 192 [60–383] ng/mL in good vs. poor outcome groups; *P* = 0.693). MMP9 levels did not correlate with the albumin index (Spearman’s rho = 0.142; *P* = 0.381). However, MMP9 levels significantly correlated with CSF glucose levels (rho = −0.419; *P* = 0.007) and admission Glasgow coma scale score (rho = 0.324; *P* = 0.032). Likewise, TIMP1 levels also did not differ by treatment outcome (1239 [889–1511] vs. 1522 [934–1949] ng/mL; *P* = 0.201). MMP9/TIMP1 ratio that reflects net proteolytic activity was also not different between the two groups (0.191 [0.107–0.250] vs. 0.163 [0.067–0.34]; *P* = 0.625).

**Conclusion:**

Our findings do not support the hypothesis that pretreatment levels of MMP9 would be higher in tuberculous meningitis patients experiencing a poor treatment outcome. Further, MMP9 levels in the CSF did not correlate with blood-brain barrier permeability in patients with tuberculous meningitis.

## Introduction

Tuberculous meningitis is the most lethal form of tuberculosis (TB), affecting about 1% of all people with TB [1]. About 40% of adults with tuberculous meningitis succumb to the disease despite early treatment with standard anti-TB treatment and corticosteroids [2]. Intensified anti-TB treatment also did not reduce the mortality in a recent clinical trial on adults with tuberculous meningitis [3]. Thus, there is a pressing need to develop host-directed therapies to improve treatment outcome in tuberculous meningitis. Of late, the role of matrix metalloproteinases (MMPs) in the pathogenesis of TB has generated considerable interest [4,5]. MMP9 plays an important role in macrophage recruitment, and destruction of extracellular matrix by MMPs could damage the blood-brain barrier (BBB) in patients with tuberculous meningitis [6,7]. Hence, MMP9 has been proposed as a potential therapeutic target in TB [4–8].

While earlier studies had found that MMP9 levels are elevated in tuberculous meningitis [9,10], very few studies have looked at the relation between the elevated MMP9 levels and treatment outcome in patients with tuberculous meningitis. Before embarking on clinical trials of MMP inhibitors such as doxycycline, it is necessary to empirically demonstrate that elevated MMP9 levels are associated with unfavorable treatment outcome. Therefore, we conducted the present study to test the hypothesis that pretreatment CSF levels of MMP9 would be higher in tuberculous meningitis patients with unfavorable treatment outcome. We also attempted to correlate the MMP9 levels with BBB permeability.

## Materials and methods

This is a case-control study nested in a treatment cohort, conducted from December 2012 to April 2014 at the Jawaharlal Institute of Postgraduate Medical Education and Research (JIPMER) hospital, Puducherry, India. The Institute Ethics Committee (Human studies) at JIPMER reviewed and approved the study protocol (approval no. IEC/SC/2012/4/127). We prospectively enrolled consecutive adolescents and adults aged >12 years admitted with a clinical diagnosis of tuberculous meningitis, after obtaining informed written consent from the patient or their guardian. We excluded patients if they did not satisfy the consensus diagnostic criteria for tuberculous meningitis [11] or tested positive for human immunodeficiency virus (HIV) infection. We excluded patients who had received anti-TB treatment or corticosteroids for more than 2 days.

We collected the following clinical and laboratory data using a standardized data collection form—demographic information, duration of symptoms, Glasgow coma scale (GCS) score, presence of focal neurological deficit, papilledema, cranial nerve palsy and BCG vaccination scar, laboratory variables such as total leukocyte count and serum albumin, and CSF analysis findings such as opening pressure, total and differential leukocyte counts, glucose, protein, Gram stain and culture, smear for acid-fast bacilli, mycobacterial culture, India ink/latex agglutination for cryptococcal antigen and adenosine deaminase (ADA) levels. We scored the severity of meningitis as per the modified British Medical Research Council (MRC) grading as follows—Grade 1, GCS score 15/15 and no focal neurological deficit; Grade 2, GCS score 15/15 with focal neurological deficit or GCS score 11–14; and Grade 3, GCS score 10 or less [12]. We performed computed-tomographic (CT) imaging of the brain at baseline in all patients, and repeated the imaging if there was any worsening of sensorium or new-onset neurological deficit. We also evaluated for evidence of TB elsewhere in the body and tested for HIV infection.

We measured the CSF opening pressure in lateral recumbent position by lumbar puncture and conventional manometry. In patients who underwent a CSF drainage procedure such as ventriculo-peritoneal shunt or external ventricular drainage before lumbar puncture, the opening pressure could not be measured, and we imputed their data with a value of 60 cm of H_2_O. In addition to routine analysis, at enrollment, we stored 2 mL of the CSF at −80°C for measurement of albumin, MMP9, and its tissue inhibitor (TIMP1) levels.

### Laboratory methods

We used commercially available solid phase sandwich ELISA kits to measure MMP9 and TIMP1 levels in the CSF (Quantikine^®^, Human MMP9 [catalog no. DMP900] and TIMP1 [catalog no. DTM100] immunoassays, R&D systems, Minneapolis, USA). The MMP9 assay measures 92 kDa pro- and 82 kDa active forms, but not the 65 kDa form of human MMP9. We tested the samples in duplicate and took the average. We measured CSF albumin levels by an immunoturbidimetric assay (MAL kits, Erba Mannheim, Germany). We calculated the albumin index (AI) as, AI = Albumin_CSF_ / Albumin_Serum_. Albumin index is a standard measure of BBB permeability [13,14]. After the conclusion of the study, we tested the remaining CSF samples for *Mycobacterium tuberculosis* by an automated cartridge-based nucleic acid amplification test (Xpert MTB/RIF, Cepheid, Sunnyvale, USA).

### Assessment of treatment outcome

We followed the patients up to the time of death, treatment completion, or study completion whichever was earlier. The last enrolled patients were followed up for a minimum of 3 months. We obtained the details of patients who died at home after hospital discharge by contacting their kin over telephone. We defined good outcome (Controls) as survival without severe neurological disability (modified Rankin scale scores of 0,1, and 2) [12,15]. We classified the remainder (death or severe neurological disability; scores 3 to 5) as poor outcome (Cases). Two observers independently scored the disability using modified Rankin scale, and resolved any disagreement by consensus before the measurement of MMP9/TIMP1 levels.

### Sample size calculation

In a previous study, the difference in MMP9 levels between patients with good vs. poor outcome was 194 ng/mL [13]. To detect a difference of this magnitude between two groups assuming a standard deviation of 186 ng/mL, with 80% power at α = 0.05 significance level, we calculated that 15 patients would be required in each group. Providing an allowance of 30% for nonparametric analysis, we required 20 patients in each group. Thus, we needed to enroll 40 patients with tuberculous meningitis in total, given that about 50% of adults would have a good treatment outcome.

### Statistical analysis

We used a statistical software package (Stata/IC 12.1 for Windows, StataCorp LP, College Station, Texas, USA) for all analyses. We divided the study sample into two groups—good and poor outcome groups. We compared the clinical and laboratory variables between good vs. poor outcome groups. We summarize normally distributed continuous variables as mean ± SD and tested between group comparisons by independent *t*-test. We present continuous variables with a skewed distribution such as MMP9 and TIMP1 levels and ordinal variables such as the GCS score as median (interquartile range, IQR) and compared them by Wilcoxon rank-sum test. We summarize categorical variables as frequency with proportion (n [%]), and compared them using Fisher’s exact test. Further, we tested the correlation of MMP9 and TIMP1 levels with albumin index and other laboratory variables by Spearman’s rank correlation. We did not adjust for multiple comparisons. We also performed a *post hoc* exploratory analysis comparing the MMP9 levels between patients that died in hospital and the rest who were alive at hospital discharge. All tests were two-sided, and a *P* < 0.05 was considered statistically significant.

## Results

**Fig 1:**
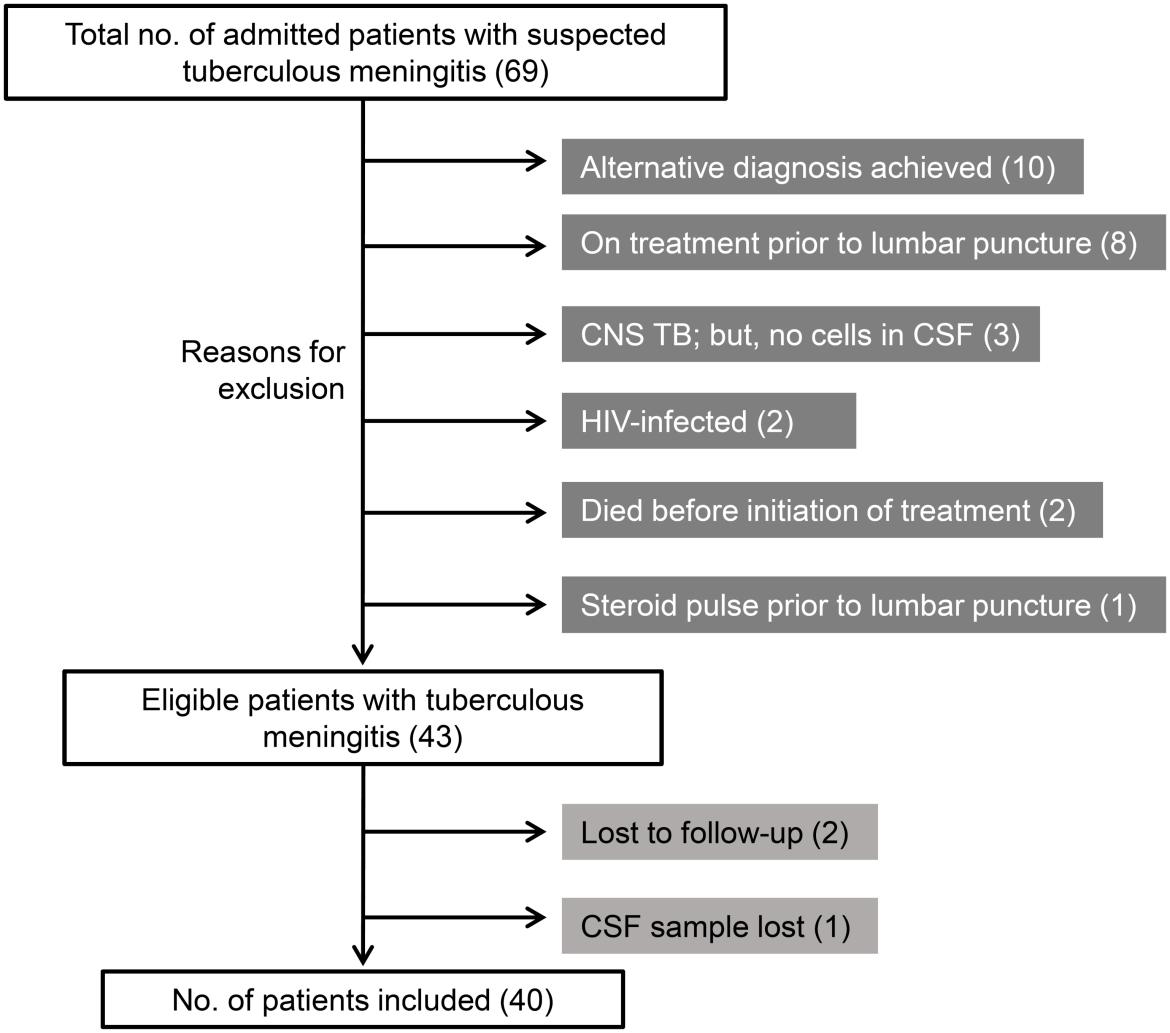
Schematic showing the enrollment of patients and reasons for exclusion. CNS = central nervous system; CSF = cerebrospinal fluid; HIV = human immunodeficiency virus; TB = tuberculosis.

We enrolled 69 patients with clinically suspected tuberculous meningitis over a period of 17 months. After excluding 29 patients for reasons listed in Fig 1, we finally included 40 patients in the present study.

### Clinical characteristics

The mean age of the patients was 36 ± 16 years. Twenty-three (58%) patients were men. Most patients presented after 2 weeks of symptom onset (median [IQR] 20 [14–60] days). Nine (23%) patients had stage 1, 15 (38%) had stage 2, and 16 (40%) had MRC stage 3 disease. In all, 17 (43%) patients required airway intubation and mechanical ventilation. About half the patients (19 [48%]) had systemic symptoms suggestive of TB, and 7 (18%) patients had had TB in the past (Table 1).

**Table 1:** Correlates of treatment outcome in 40 patients with tuberculous meningitis.

Characteristic	All patients (N = 40)	Good outcome (n = 22)	Poor outcome (n = 18)	*P-*value
Age, years^a^	36 ± 16	33 ± 13	40 ± 17	0.169
Male gender	23 (58)	10 (45)	13 (72)	0.088
Duration of symptoms, days^b^	20 (14–60)	20 (14–60)	20 (10–60)	0.682
Seizures	10 (25)	3 (14)	7 (39)	0.140
TB in the past	7 (18)	4 (18)	3 (17)	1.000
BCG scar	23 (62)	14 (67)	9 (56)	0.517
GCS score^b^	12 (9–14)	14 (11–15)	9 (8–11)	0.001
Focal neurological deficit	9 (23)	4 (18)	5 (28)	0.705
Cranial nerve palsy	15 (38)	7 (32)	8 (45)	0.412
MRC Grade 1 Grade 2 Grade 3	9 (23) 15 (38) 16 (40)	7 (32) 10 (45) 5 (23)	2 (11) 5 (28) 11 (61)	0.05
Hydrocephalus	17 (43)	8 (36)	9 (50)	0.385
CSF drainage procedures	12 (30)	4 (18)	8 (44)	0.093
Time to treatment initiation, days^b^	4 (2–5)	3 (1–5)	4 (2–6)	0.26
Blood hemoglobin, g/dL^a^	11.93 ± 2.2	10.8 ± 1.7	13.3 ± 1.9	< 0.001
Total leukocyte count, cells/μL^a^	10030 ± 4196	8668 ± 2867	11694 ± 4990	0.031
CSF opening pressure, cm of H_2_O^b^	30 (16–48)	25 (16–40)	39 (25–58)	0.115
CSF leukocyte count, cells/μL^b^	190 (75–315)	160 (70–280)	200 (80–330)	0.540
CSF lymphocyte percentage^b^	73 (30–90)	69 (44–89)	78 (23–90)	0.795
CSF protein, mg/dL^b^	151 (114–220)	140 (116–171)	158 (98–225)	0.471
CSF glucose, mg/dL^b^	29 (21–41)	29 (17–39)	29 (21–47)	0.807
CSF adenosine deaminase, U/L^b^	14 (9–19)	11 (9–16)	17 (14–22)	0.091
CSF albumin, mg/L^a^	448 ± 120	477 ± 83	412 ± 147	0.106
Serum albumin, g/dL^a^	3.6 ± 0.62	3.7 ± 0.6	3.4 ± 0.50	0.116
Albumin index^a^	0.013 ± 0.004	0.0132 ± 0.0031	0.0125 ± 0.0050	0.607

^a^ data presented as mean ± SD;

^b^ data presented as median (IQR); all other data presented as n (%).

BCG = bacille Calmette-Guerin; CSF = cerebrospinal fluid; GCS = Glasgow coma scale; MRC = Medical Research Council; TB = tuberculosis

CT scan of the brain was abnormal in 28 (70%) patients—17 (43%) had hydrocephalus; 9 (23%) had basal exudates; 8 (20%) had tuberculomas; and 5 (13%) patients had infarcts. Chest radiographic abnormalities were found in 18 (45%) of 40 patients; 6 (15%) had miliary nodules; and 1 patient had bilateral exudative, lymphocytic pleural effusions with high ADA level. High-resolution CT scan of the chest showed findings suggestive of TB in 5 patients. In total, 19 (48%) patients had evidence of TB elsewhere—18 patients had chest radiographic features of TB; 2 patients had TB lymphadenitis (in one it was confirmed by lymph node biopsy which showed caseating granulomatous inflammation; in the other patient, necrotic lymph nodes were present in the neck, mediastinum and abdomen on imaging); and 1 patient each had encysted ascites and Pott’s spine (numbers not mutually exclusive). *M*. *tuberculosis* was detected in CSF by Xpert MTB/RIF in 10 patients. Rifampicin-resistance was not identified in any of the positive samples. Of the 40 patients, 10 (25%) had definite, 19 (48%) had probable and 11 (28%) had possible tuberculous meningitis as per the consensus case-definition.

### Treatment outcome

All patients received first-line anti-TB drugs—isoniazid (H), rifampicin (R), pyrazinamide (Z), ethambutol (E) and/or streptomycin (S) along with pyridoxine supplementation. The median time to treatment initiation was 4 (2–5) days. Treatment was started after 1 week in 5 patients. Fifteen (38%) patients received HRZE regimen; 22 (55%) received HRZS regimen; and the remaining 3 patients received HRZES regimen during the intensive phase of treatment. All patients except one received adjunctive dexamethasone.

The median duration of follow-up was 106 (range 5–365) days. Eleven (28%) patients died in hospital. Of the 29 patients discharged alive from the hospital, 4 patients died at home—3 of them died within 1 month and 1 patient died after 3 months of hospital discharge. The total mortality in the study sample was 15 (38%). Of the 25 patients that were alive at study completion, 3 (18%) patients had persistent neurological disability. Thus, the outcome was poor in 18 (45%) patients in total; 22 (55%) patients had a good outcome—4 were totally asymptomatic, 7 had a modified Rankin score of 1, and 11 patients had a score of 2 (S1 Fig).

### Clinical correlates of treatment outcome

On comparison, lower GCS score at presentation was significantly associated with a poor outcome (*P* = 0.001; Table 1). Most patients with a poor outcome had MRC grade 3 meningitis at presentation, whereas a little less than a fourth of patients with good outcome had grade 3 disease (*P* = 0.05). Blood hemoglobin concentration and total leukocyte count were significantly higher in patients with poor outcome (Table 1). Although the CSF opening pressure was higher in patients with poor outcome, the difference was not statistically significant. There were no significant differences between the two groups with respect to CSF characteristics such as albumin index, total leukocyte count, glucose and protein levels. Median time to treatment initiation was similar in the two groups (Table 1).

### Correlation of MMP9 and TIMP1 with CNS inflammation

**Fig 2:**
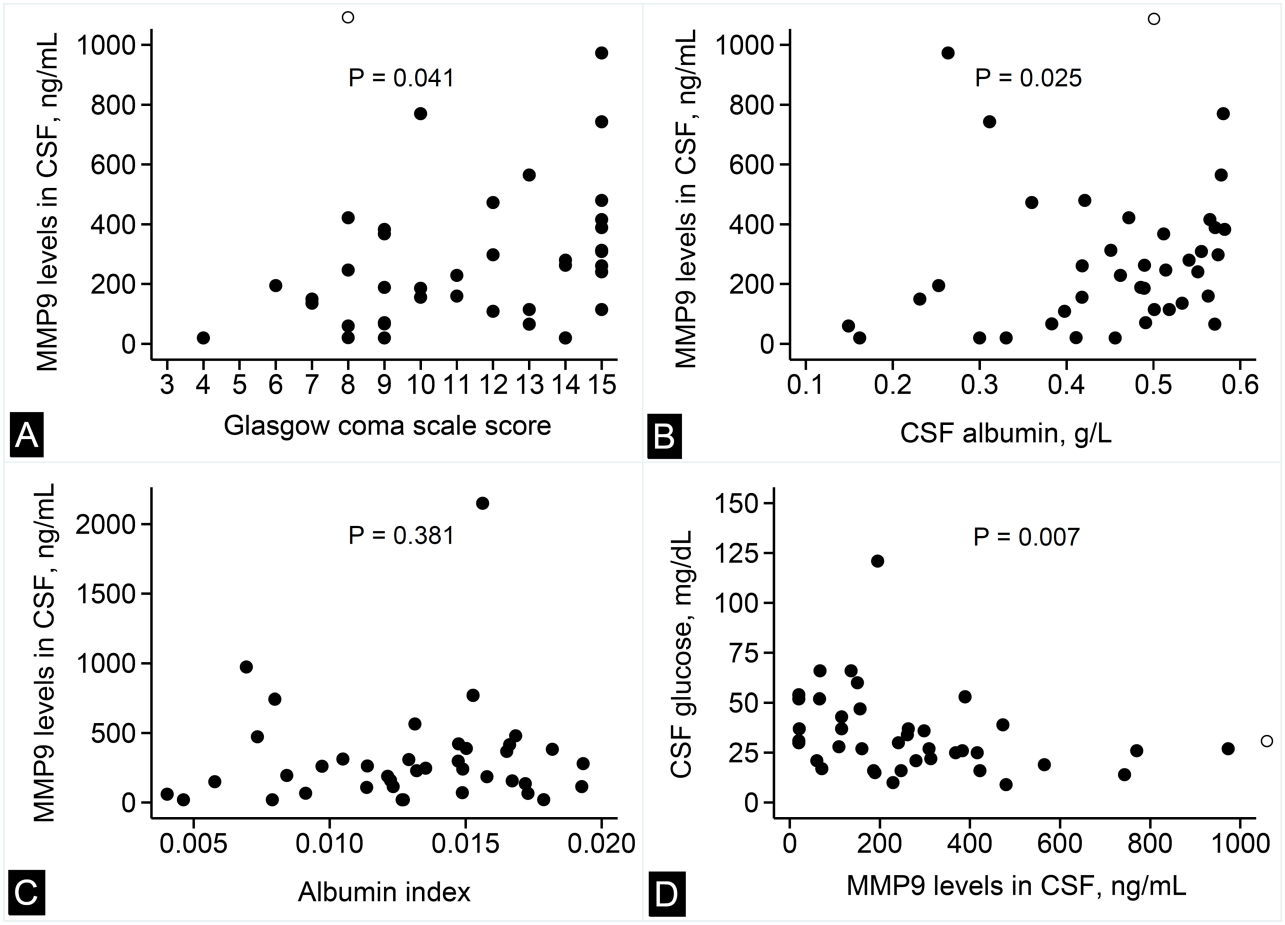
Correlation of MMP9 levels with clinical and laboratory variables. Scatter plots showing the correlation between MMP9 in the CSF with Glasgow coma scale score (panel A), CSF albumin levels (panel B), albumin index (panel C), and CSF glucose levels (panel D). In panels A,B and D, the hollow circle indicates an extreme value of CSF MMP9 level (2150 ng/mL). *P*-values are for Spearman’s rank correlation.

MMP9 levels in the CSF had a significant positive correlation with the GCS score (Spearman’s rho = 0.324; *P* = 0.041; Fig 2A) and CSF albumin levels (Spearman’s rho = 0.354; *P* = 0.025; Fig 2B). However, the MMP9 levels did not correlate with albumin index (Spearman’s rho = 0.142; *P* = 0.381; Fig 2C). The MMP9 levels showed a significant negative correlation with CSF glucose levels (Spearman’s rho = −0.419; *P* = 0.007; Fig 2D). Further, MMP9 levels did not correlate with neutrophil counts in the CSF (Spearman’s rho = 0.133; *P* = 0.413; S2 Fig).

There was no apparent correlation between MMP9 and TIMP1 levels in the CSF (Spearman’s rho = 0.149; *P* = 0.358; S3 Fig). TIMP1 levels positively correlated with CSF protein (Spearman’s rho = 0.342; *P* = 0.031; S4 Fig) and ADA levels (Spearman’s rho = 0.327; *P* = 0.048; S5 Fig). Although the CSF opening pressure did not show a significant correlation with MMP9 levels or albumin index, it correlated positively with the CSF neutrophil count (Spearman’s rho = 0.368; *P* = 0.03; S6 Fig).

### MMP9, TIMP1, and treatment outcome

**Fig 3:**
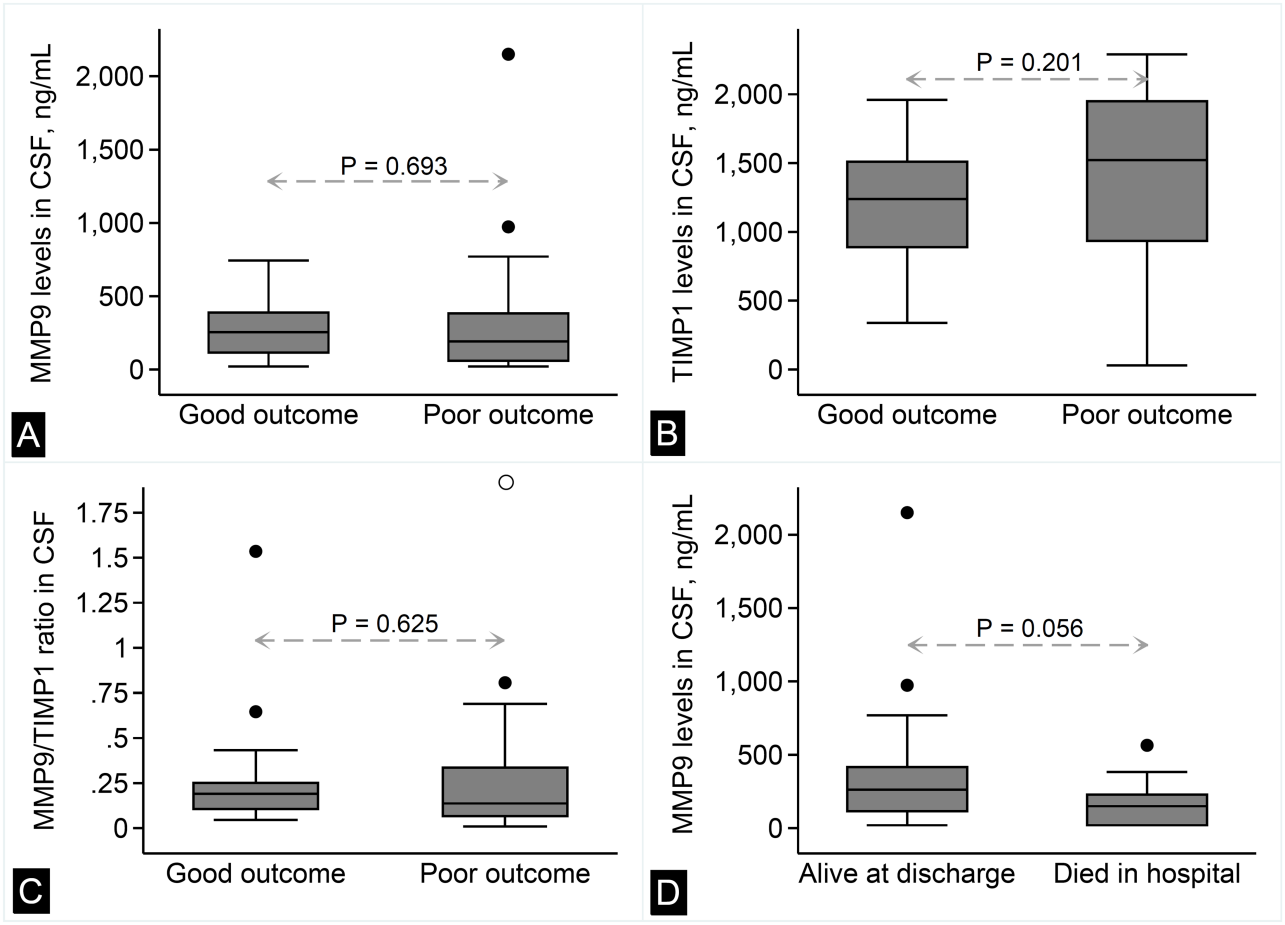
Treatment outcome and CSF levels of MMP9, TIMP1, and MMP9/TIMP1 ratio. Box-whisker plots showing the comparison of MMP9 levels (panel A), TIMP1 levels (panel B), and MMP9/TIMP1 ratio (panel C) between tuberculous meningitis patients with good vs. poor treatment outcome. Panel D depicts the comparison of MMP9 levels between patients that died in hospital and the rest. *P*-values are for Wilcoxon rank-sum test.

The median MMP9 level in the CSF was 254 (115–389) ng/mL in patients with good outcome and 192 (60–383) ng/mL in patients with poor outcome. This difference, however, was not statistically significant (Fig 3A; *P* = 0.693). Similarly, there was no significant difference in TIMP1 level between the two groups (Fig 3B; 1239 [889–1511] vs. 1522 [934–1949] ng/mL; *P* = 0.201). MMP9/TIMP1 ratio that reflects net proteolytic activity was also not different between the two groups (Fig 3C; 0.191 [0.107–0.250] vs. 0.163 [0.067–0.34]; *P* = 0.625). On exploratory analysis, the apparently lower level of MMP9 in patients with poor outcome was more pronounced when patients that died in hospital (early deaths) were compared to the rest (150 [20–229] vs. 263 [115–416]; *P* = 0.056; Fig 3D).

## Discussion

We found that tuberculous meningitis patients with a poor treatment outcome did not have higher MMP9 levels in the CSF at baseline. The MMP9 levels did not correlate with markers of BBB permeability such as albumin index and CSF opening pressure. Instead, MMP9 and TIMP1 levels nonspecifically correlated with markers of intrathecal inflammation such as CSF glucose, protein, and ADA levels. We also found that early deaths were associated with a lower level of MMP9, which was quite contrary to our study hypothesis.

There are only a handful of published studies on MMP9 levels in patients with tuberculous meningitis. Rom and colleagues reported for the first time in 1996 that *M*. *tuberculosis* upregulated the expression of MMP9 [16]. In the year 2000, Matsurra et al reported that CSF MMP9 levels were elevated in tuberculous meningitis (n = 7) as compared to aseptic meningitis (n = 9), and patients with severe disease had higher levels of MMP9 [9]. Subsequently, Lee et al confirmed that MMP9 levels were significantly higher in tuberculous meningitis (n = 24) even after Day 7 of treatment, and higher levels were associated with neurological complications [10]. However, both these studies did not follow a standard definition of poor outcome. Price et al reported in 2001 that MMP9 concentration per CSF leukocyte was significantly higher in fatal tuberculous meningitis (n = 23) [7]. Further, patients with low GCS score, confusion, or focal neurological deficit had significantly higher levels of MMP9 per CSF leukocyte.

However, a subsequent study from the same setting (n = 21) did not find a statistically significant difference in CSF MMP9 levels between survivors and the rest [13]. On the other hand, in a sub-study of a large clinical trial again from the same setting, patients treated with adjunctive dexamethasone (n = 11) had a significant fall in MMP9 levels by Day 5 of treatment [17]. In the absence of any demonstrable effect on intrathecal immune response [18], the authors ascribed the efficacy of dexamethasone to the fall in MMP9 levels. In an *ex vivo* experimental model, MMP blockade prevented BBB disruption by *M*. *tuberculosis* [19]. Notwithstanding, we found that MMP9 levels did not correlate with albumin index, a standard *in vivo* measure of BBB permeability. Previously, Thwaites et al and Green et al have also documented a lack of association between MMP9 levels and albumin index *in vivo* [13,17]. Thus, taken together, available evidence on the relation between MMP9 levels and treatment outcome in tuberculous meningitis is equivocal. Further, previous studies were limited by small sample sizes and insufficient number of patients experiencing unfavorable outcome. Moreover, these studies did not examine the clinically relevant outcome of survival without severe neurological disability, an outcome which has been empirically validated in multiple randomized controlled trials [2,12].

Very recently, a study of 36 HIV-negative tuberculous meningitis patients has been reported from northern India [20]. In this study, MMP9 levels were not associated with treatment outcome similar to ours. However, for unclear reasons, the median MMP9 levels reported in this study were much lower than our findings (4962 pg/mL vs. 235 ng/mL). Moreover, this study did not assess TIMP1 levels and BBB permeability. MMP9 levels observed in our study are comparable to earlier reports by Thwaites et al and Green et al [13,17].

The present study has several strengths—first, it was larger than previous studies, with sufficient number of patients in good as well as poor outcome groups; second, we studied the clinically relevant outcome of survival without neurological disability, and two observers independently scored the disability before estimating MMP9/TIMP1 levels to eliminate bias; third, we followed the recently proposed international consensus case-definition, in order to minimize difficulties in diagnosing tuberculous meningitis; and finally, we also studied the relationship between MMP9 levels and BBB permeability.

On the other hand, there are three possible limitations to this study. First, we imputed the CSF opening pressure values in patients that underwent a CSF drainage procedure before lumbar puncture. This was necessary from a safety perspective, and it seems logical to presume that such patients would have had high opening pressures. Second, drug-resistance could have confounded our results. However, no case of rifampicin-resistance was identified in this study, and as such multidrug-resistance is uncommon among tuberculous meningitis patients in India [21]. Third, we could have used gelatin zymography or a fluorogenic substrate-based assay, which also detect lower molecular weight isoforms of MMP, to measure enzyme activity levels in the CSF and to differentiate active MMP from pro-MMP. We estimated MMP9 levels by ELISA to enable comparison with previous pivotal studies which had used this assay [13,17].

One possible explanation for the lack of association between MMP9 levels and treatment outcome could be that not all deaths in tuberculous meningitis are attributable to CNS inflammation. Of note, in the present study, 4 of the 15 deaths occurred after hospital discharge. It is possible that these late deaths were attributable to poor CNS penetration by anti-TB drugs rather than CNS inflammation. However, our exploratory analysis looking at in-hospital deaths (early deaths) does not lend support to this explanation.

Apart from failing to prove the study hypothesis, the present study has two other important findings. First, the elevated MMP9 levels appear to be a nonspecific reflection of intrathecal inflammation as evidenced by the correlation of MMP9 and TIMP1 levels with CSF glucose, albumin, and ADA levels. The natural history of CSF inflammation in untreated tuberculous meningitis is characterized by progressive increase in protein levels accompanied by decreasing glucose levels on serial analysis [22,23]. Longitudinal changes in CSF protein, glucose, and possibly MMP9 and TIMP1 levels prior to treatment might yield spurious correlations between these variables on a cross-sectional analysis. Although statistically significant, such correlations may not have any biological significance. Second, the current finding that higher MMP9 levels were associated with better GCS scores and in-hospital survival is counterintuitive. Proving the association between elevated levels of MMP9 and treatment outcome is important to justify a clinical trial of adjunctive treatment with already licensed drugs having MMP-inhibitory activity, such as doxycycline. In fact, several authors have made a case for doxycycline as adjunctive treatment in TB [4,6,8,24]. However, our findings temper this enthusiasm, at least in the case of tuberculous meningitis.

One could reasonably argue that it is not necessary to demonstrate a difference in MMP9 levels, since MMP9 is anyway damaging to the tissues. However, the association of higher MMP9 levels with better GCS scores suggests a possibility that MMP9 inhibition could be counterproductive, since altered consciousness is an important prognostic factor in tuberculous meningitis [25,26]. Notwithstanding the fact that MMPs disrupt the BBB, biologically the association of elevated MMP9 levels with better survival is not implausible—a leaky BBB could enhance tissue penetration of anti-TB drugs, many of which poorly penetrate the BBB otherwise [27]. Hence, before embarking on a clinical trial of MMP inhibitors in tuberculous meningitis, it would be prudent to refute or confirm the present findings in a larger cohort of patients with tuberculous meningitis.

## Conclusions

We found that pretreatment MMP9 levels were not associated with treatment outcome in patients with tuberculous meningitis. Moreover, we did not find any correlation between MMP9 levels and BBB permeability. On the other hand, MMP9 and TIMP1 levels showed nonspecific correlation with some CSF inflammation variables. Hence, further studies are warranted to justify a clinical trial of MMP9 inhibitors as adjunctive treatment in tuberculous meningitis, which remains the most lethal form of TB.

## Supporting information

S1 Fig Vital status and functional outcome in 40 patients with tuberculous meningitis. (JPG)

S2 Fig Correlation of MMP9 levels with neutrophil counts in the CSF. (PDF)

S3 Fig Correlation between MMP9 and TIMP1 levels in the CSF. (PDF)

S4 Fig Correlation of TIMP1 levels with CSF protein levels. (PDF)

S5 Fig Correlation of TIMP1 levels with CSF adenosine deaminase levels. (PDF)

S6 Fig Correlation between CSF opening pressure and CSF neutrophil counts. (PDF)
