# Supplementary figures and images for: Cerebrospinal fluid matrix metalloproteinase 9 levels, blood-brain barrier permeability, and treatment outcome in tuberculous meningitis
Source: PLoS One. 2017 Jul 12;12(7):e0181262. doi: 10.1371/journal.pone.0181262 (PMC5507543; doi:10.1371/journal.pone.0181262)

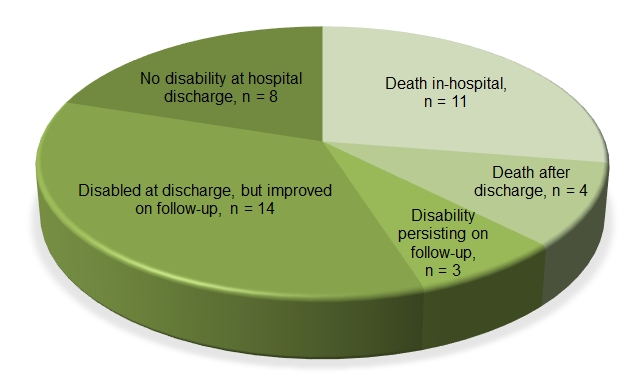

Supplement: S1 Fig — (JPG) [file pone.0181262.s001.jpg]

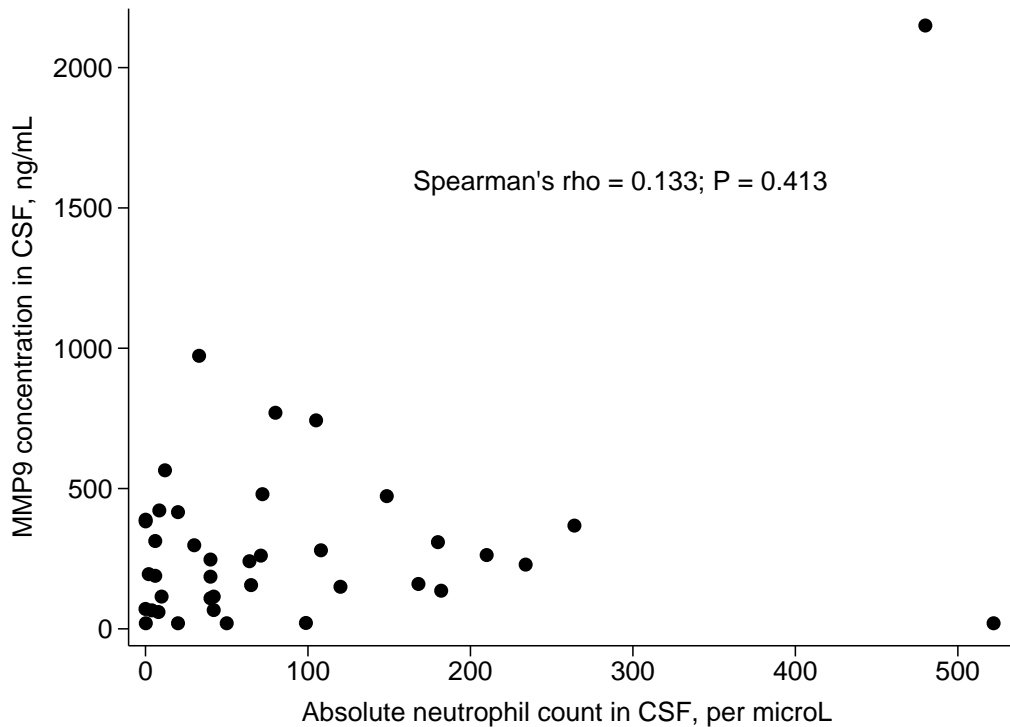

Supplement: S2 Fig — (PDF) [file pone.0181262.s002.pdf]

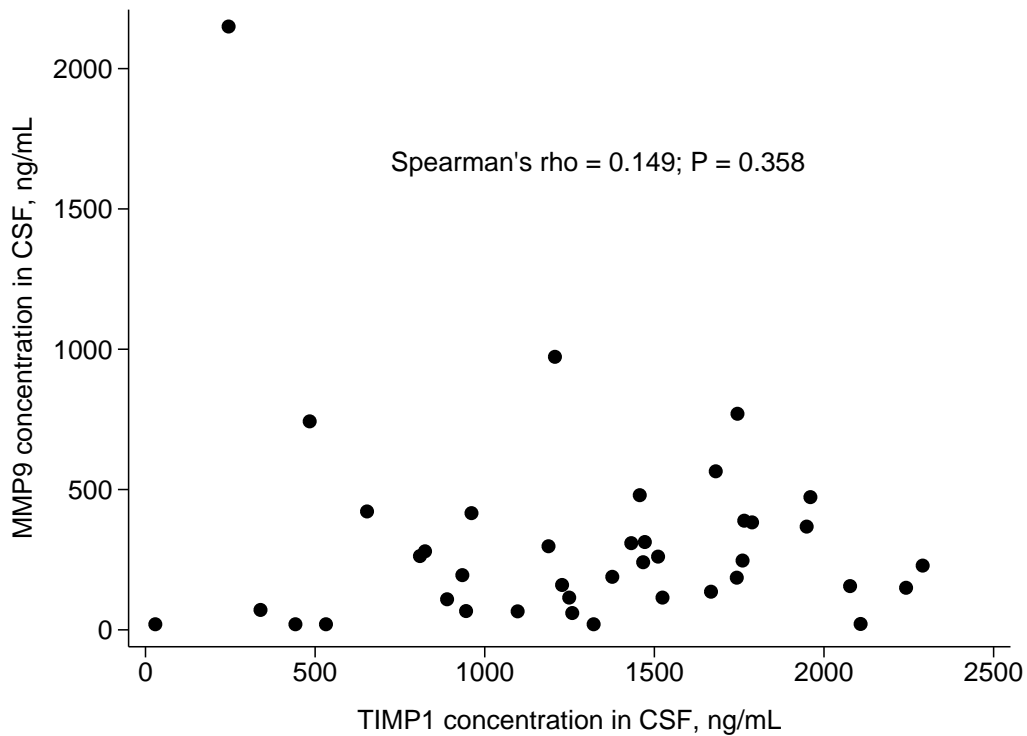

Supplement: S3 Fig — (PDF) [file pone.0181262.s003.pdf]

TIMP1 concentration in CSF, ng/mL

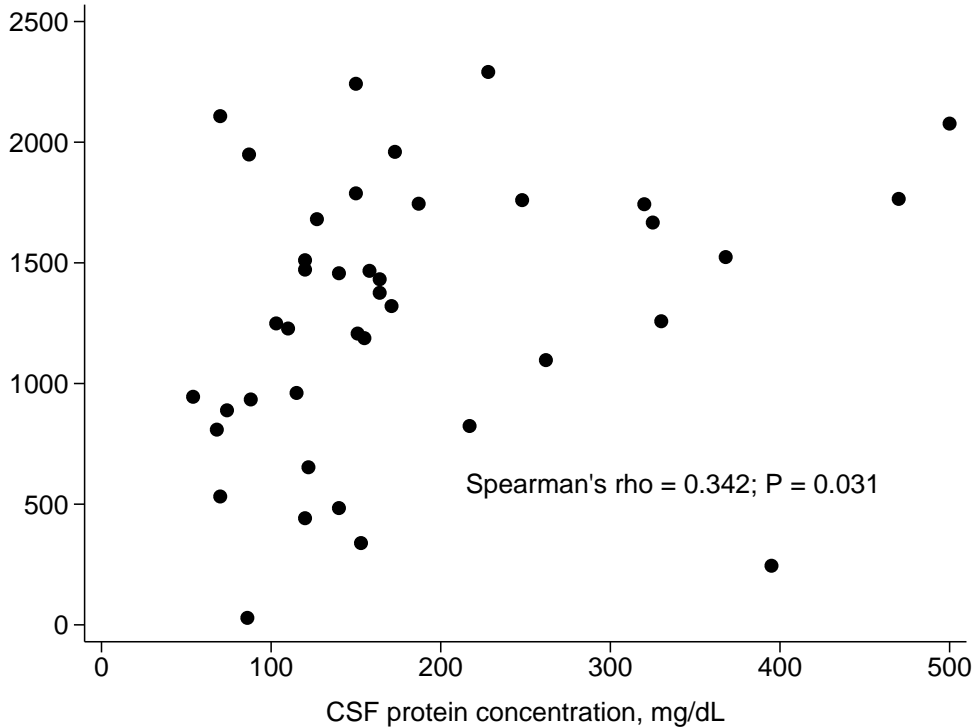

Supplement: S4 Fig — (PDF) [file pone.0181262.s004.pdf]

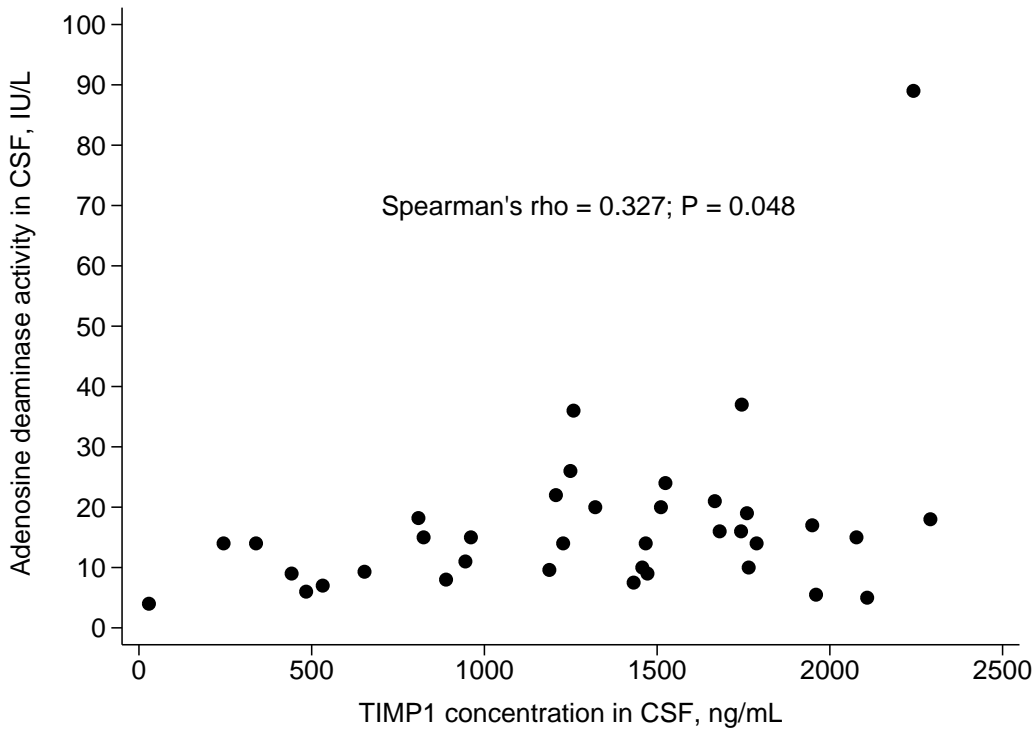

Supplement: S5 Fig — (PDF) [file pone.0181262.s005.pdf]

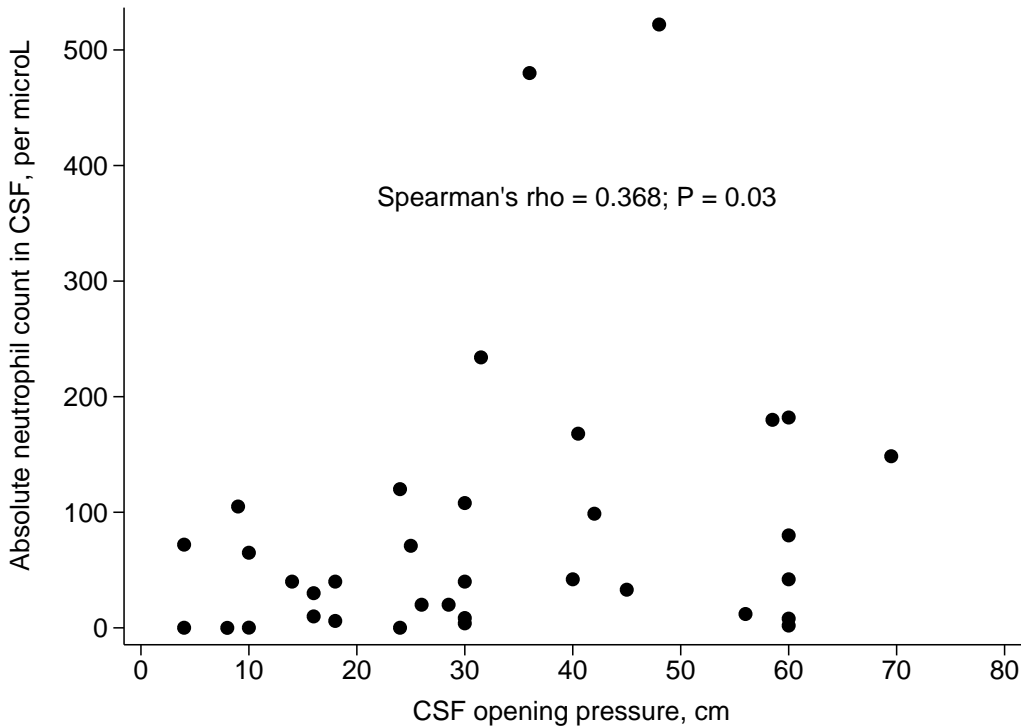

Supplement: S6 Fig — (PDF) [file pone.0181262.s006.pdf]
